# Small-Molecule Inhibitors of Amyloid Beta: Insights from Molecular Dynamics—Part A: Endogenous Compounds and Repurposed Drugs

**DOI:** 10.3390/ph18030306

**Published:** 2025-02-23

**Authors:** Mariyana Atanasova

**Affiliations:** Faculty of Pharmacy, Medical University of Sofia, 1000 Sofia, Bulgaria; matanasova@pharmfac.mu-sofia.bg

**Keywords:** amyloid beta, amyloidosis, molecular dynamics (MD), Alzheimer’s disease (AD), amyloid beta inhibitors

## Abstract

The amyloid hypothesis is the predominant model of Alzheimer’s disease (AD) pathogenesis, suggesting that amyloid beta (Aβ) peptide is the primary driver of neurotoxicity and a cascade of pathological events in the central nervous system. Aβ aggregation into oligomers and deposits triggers various processes, such as vascular damage, inflammation-induced astrocyte and microglia activation, disrupted neuronal ionic homeostasis, oxidative stress, abnormal kinase and phosphatase activity, tau phosphorylation, neurofibrillary tangle formation, cognitive dysfunction, synaptic loss, cell death, and, ultimately, dementia. Molecular dynamics (MD) is a powerful structure-based drug design (SBDD) approach that aids in understanding the properties, functions, and mechanisms of action or inhibition of biomolecules. As the only method capable of simulating atomic-level internal motions, MD provides unique insights that cannot be obtained through other techniques. Integrating experimental data with MD simulations allows for a more comprehensive understanding of biological processes and molecular interactions. This review summarizes and evaluates MD studies from the past decade on small molecules, including endogenous compounds and repurposed drugs, that inhibit amyloid beta. Furthermore, it outlines key considerations for future MD simulations of amyloid inhibitors, offering a potential framework for studies aimed at elucidating the mechanisms of amyloid beta inhibition by small molecules.

## 1. Introduction

Dementia is a term associated with memory loss, difficulties in thinking, learning, judgment, and social abilities. It ranks seventh among the leading causes of death across all age groups and fourth among elderly individuals over 70 years old [1]. According to the WHO, as of 2023, more than 55 million people worldwide are living with dementia, with this number increasing by 10 million annually [2]. Alzheimer’s disease (AD) is the most prevalent form of dementia, accounting for 60–70% of cases globally [2]. Additionally, AD is the most common form of amyloidosis, a group of diseases characterized by the abnormal deposition of fibrillar proteins [3]. The pathological hallmarks of AD include senile plaques—extracellular deposits of amyloid β (Aβ) peptide—and intracellular neurofibrillary tangles (NFTs) composed of hyperphosphorylated tau (τ) protein. Moreover, the complexity of the disease involves oxidative stress, which triggers inflammation and leads to Aβ- and tau-induced neurodegeneration [4,5]. Other key factors include the low levels of the neurotransmitter acetylcholine (ACh) in synaptic junctions, resulting in cognitive dysfunction, as well as synaptic and neuronal loss [6,7,8]. AD primarily affects cortical and hippocampal neurons in the human brain. One current therapeutic approach is based on the cholinergic hypothesis, which involves inhibiting the enzyme acetylcholinesterase (AChE) to increase ACh levels, thereby improving cholinergic function and cognition [9,10,11]. Another approved treatment targets overstimulated N-methyl-D-aspartate (NMDA) receptors through the antagonist memantine, which reduces hyperexcitability and prevents neuronal death [4]. However, these treatments are only symptomatic and do not prevent disease progression. As a result, alternative strategies for discovering new anti-AD agents—such as those based on the amyloid cascade and tau hypotheses—are currently the focus of research [12,13,14,15,16]. Despite extensive studies, the pathogenesis of the disease remains to be fully elucidated.

This review is organized into three main sections. The first section examines the amyloid hypothesis along with the structural characteristics and forms of peptide monomers, dimers, oligomers, and their stabilizing interactions. The second section focuses on molecular dynamics simulations, addressing challenges related to timescale limitations and sampling deficiencies. Finally, the third section summarizes and discusses molecular dynamics studies from the past decade on small molecules, specifically endogenous compounds and repurposed drugs, that inhibit amyloid beta.

## 2. Amyloid Hypothesis

**Figure 1:**
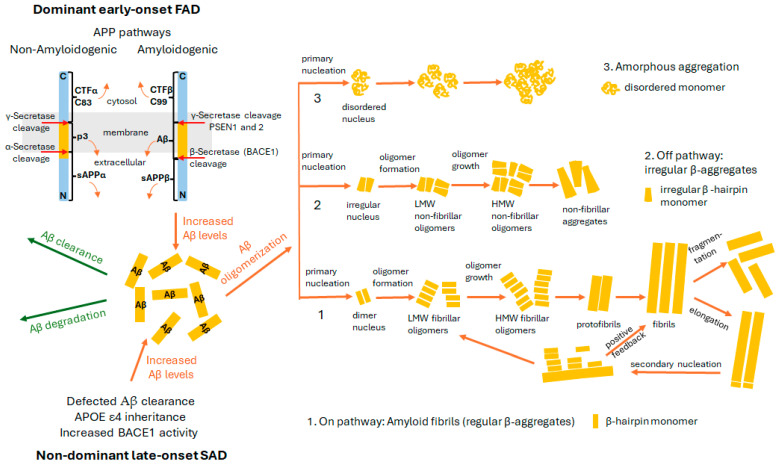
The factors contributing to elevated Aβ levels in both forms of Alzheimer’s disease (AD) are illustrated on the left. Mutations in the APP gene, as well as in the PSEN1 and PSEN2 genes, which encode the catalytic subunits (presenilins 1 and 2) of γ-secretase, inheritance of the APOE ε4 allele, increased activity of β-secretase (BACE1), and impaired Aβ clearance all contribute to Aβ accumulation, reinforcing the amyloid cascade hypothesis The APP cleavage pathways are shown schematically. In the non-amyloidogenic pathway, the cleavage by α-secretase and γ-secretase generates soluble APPα (sAPPα), p3 (composed of 17–40/42 amino acids), and the C-terminal fragment α (CTFα, C83), containing 83 amino acids. In the amyloidogenic pathway, β-secretase first cleaves APP, resulting in sAPPβ, and subsequently, γ-secretase generates Aβ (1–40/42 amino acids) and CTFβ (C99), which consists of 99 amino acids. The increased levels of Aβ can be cleared, degraded, or undergo oligomerization. Based on Aβ’s secondary structure, three potential self-aggregation pathways via primary nucleation have been identified: specific on-pathways, off-pathways, and a non-specific amorphous pathway. Amyloid fibrils are formed from the primary nucleation of U-shaped β-strand (β-hairpin) monomers, which then aggregate into a nucleus, low- and high-molecular-weight fibrillar oligomers, and amyloid protofibrils (pathway 1). Alternatively, an irregular β-sheet monomer self-associates through an irregular nucleus and oligomers, eventually forming non-toxic aggregates through the off-pathway (pathway 2). The third pathway leads to the creation of amorphous, unstructured aggregates (tangles) composed of disordered random coil monomers. Secondary mechanisms for aggregate formation, depending on fibril concentration, are also outlined, including fibril fragmentation (monomer-independent), elongation, and secondary nucleation (monomer-dependent).

The amyloid hypothesis currently serves as the leading model of AD pathogenesis [12]. According to this hypothesis, Aβ peptide is the central factor that induces neurotoxicity and triggers of pathological events within the central nervous system (CNS). The primary pathological change observed in the AD brain is the aggregation of Aβ into oligomers and deposits, which initiate processes such as vascular damage, activation of astrocytes and microglia due to inflammation, and disruption of neuronal ionic homeostasis and oxidative stress. These changes lead to altered kinase or phosphatase activities, tau phosphorylation, neurofibrillary tangle (NFT) formation, cognitive decline, synaptic loss, cell death, and dementia [12,17]. Additionally, it has been observed that toxic phosphorylated tau forms exacerbate Aβ toxicity through a feedback loop [18]. The amyloid hypothesis is further supported by the mutations in the APP (amyloid precursor protein) gene, as well as in the PSEN1 and PSEN2 genes, which encode the catalytic subunits of γ-secretase [19,20]. These mutations are associated with dominant, early-onset familial AD (FAD). Furthermore, non-dominant, late-onset sporadic AD (SAD) is linked to genetic factors such as the APOE ε4 (apolipoprotein E ε4) allele [21,22,23], as well as issues with Aβ clearance [24] and increased BACE-11 (β—secretase 1, β-site amyloid precursor protein cleaving enzyme 1) activity [25] (Figure 1). The Aβ peptide is produced through the cleavage of the amyloid-β precursor protein, APP. There are three major forms of APP produced by the APP gene, which vary in length and location. These include APP695, consisting of 695 residues and primarily produced in neurons; APP751, composed of 751 amino acids (aa) and expressed in endothelial cells; and APP770, made up of 770 aa and found in platelets [26]. APP is a type I transmembrane glycoprotein composed of three domains: (i) an extracellular glycosylated N-terminal ectodomain (residues 18–624), (ii) a single hydrophobic transmembrane domain (TMD) (residues 625–648), and (iii) a short intracellular domain (AICD) (residues 649–695) [27,28,29,30]. APP undergoes two proteolytic cleavage pathways (Figure 1). In the non-amyloidogenic pathway, cleavage occurs in two steps, first by α-secretase and then by γ-secretase. The α-secretase cleavage results in the release of the soluble ectodomain sAPPα into the extracellular space. The remaining C-terminal fragment, CTFα (83 amino acids), remains embedded in the membrane, where γ-secretase cleaves it, producing the fragment p3 and the APP intracellular domain (AICD), which are released into the extracellular space and cytoplasm, respectively. In the amyloidogenic pathway, β-secretase cleaves APP to generate sAPPβ, which is released into the extracellular space, while the C99 (CTFβ) fragment remains anchored in the membrane. Next, γ-secretase cleaves C99 into Aβ, which is released from the cell, while the AICD is released into the cytosol (Figure 1) [23,31,32]. The Aβ molecule is a 4 kDa polypeptide consisting of 38–53 amino acids [33]. Among the different Aβ isoforms, Aβ_1–42_ is considered the most neurotoxic [34,35], making it the primary focus of this review. It undergoes sequential self-aggregation, forming various structures such as soluble oligomers, insoluble protofibrils, and fibrils through a primary nucleation mechanism. Three potential pathways for the self-aggregation of Aβ have been proposed, based on the initial secondary structure of Aβ_1–42_ (Figure 1) [36,37]. The toxic amyloid pathway begins with the primary nucleation of U-shaped β-strand monomers into nuclei during the lag phase. A key early step in amyloidogenesis is the conformational conversion from an α-helix or random coil structure to a β-sheet structure During the second growth phase, soluble oligomers and protofibrils with molecular weights of approximately 20 kDa (1–2 nm) (low MW) for tetramers and 56–60 kDa (3–5 nm) (high MW) for dodecamers are formed, respectively [38]. Finally, in the stationary phase, amyloid fibrils are formed [39]. Additionally, fibrils can form aggregates through secondary mechanisms, which can be classified based on their dependence or independence on monomer concentration, such as elongation and secondary nucleation (dependent) or fragmentation (independent) (Figure 1) [40,41,42]. In secondary nucleation, the surfaces of fibrils catalyze the formation of new low-molecular-weight (LMW) oligomers. Once a critical fibril concentration is reached, this mechanism becomes the primary source of oligomers, surpassing the primary nucleation process [40]. This positive feedback leads to the rapid, exponential accumulation of toxic oligomers [43,44,45]. Recent findings have shown that the severity of the disease correlates with the levels of soluble Aβ oligomers [46,47], which are recognized as the main neurotoxic agents. These oligomers disrupt various cellular processes through multiple mechanisms [39,48,49]. They can bind to numerous membrane receptors, disrupting normal signaling pathways, and also form pore-like structures in the neuronal membrane, altering its permeability and integrity (as reviewed in [39]). As a result, the dysregulation of Ca^2+^ homeostasis, mitochondrial dysfunction, reactive oxygen species (ROS) generation, reduced ATP levels, and tau phosphorylation occurs, ultimately leading to synaptic dysfunction and neuronal loss. Consequently, the amyloid cascade hypothesis has been revised to the “oligomer hypothesis” [50].

Based on the current understanding of amyloidogenesis, researchers are focusing on several strategies for treating AD. These strategies include the following: (i) Reducing Aβ production through α-secretase activators, and β-secretase (BACE1) and γ-secretase inhibitors. (ii) Enhancing Aβ clearance via active or passive immunization, receptor-mediated clearance, modulation of APP-tau interaction, and targeting Aβ and neuroinflammation. A significant breakthrough was achieved through the passive immunization approach, which involves the direct administration of antibodies. This method has proven to be highly effective, with the antibodies being well tolerated and considered safe due to their high specificity [51,52]. Two monoclonal antibodies, Aducanumab and Lecanemab, received approval from the US Food and Drug Administration (FDA) on 7 July 2021, and 22 January 2023, respectively, for the treatment of AD [53,54]. (iii) Inhibiting Aβ oligomerization with amyloid inhibitors, which can be categorized into various types: small organic molecules (both natural and synthetic), carbon-based nanomaterials (such as fullerenes, carbon nanotubes, graphene oxide, and carbon dots), nanoparticles (including polymer, metallic, and lipid-based types), and metal chelators (such as iron, copper, and zinc ion chelators). For more information on AD treatment strategies, see references [15,51,52,53,54,55,56,57]. An underexplored approach in studying and understanding the molecular mechanisms of oligomer formation, the kinetics of aggregation, and designing potential modulators of fibrillogenesis involves promoters, rather than inhibitors, of Aβ aggregation [58,59,60,61,62,63,64].

## 3. Structural Characteristics and Forms of Monomers, Dimers, and Oligomers

**Figure 2:**
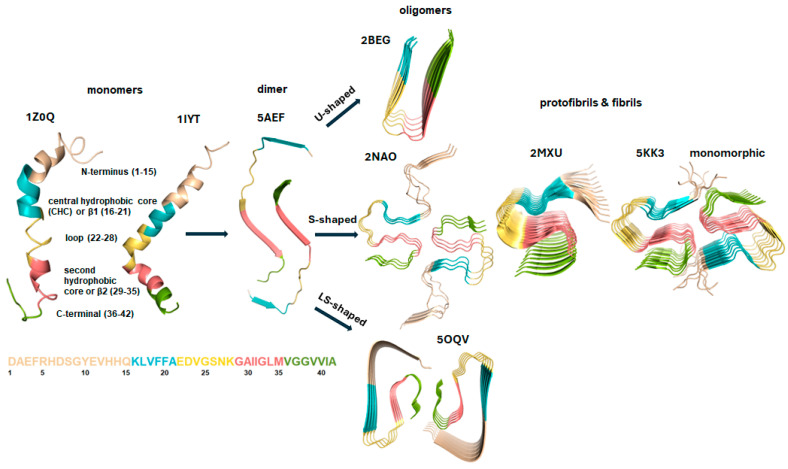
The primary and secondary structures of human Aβ_1–42_ the known aggregate shapes, as retrieved from the Protein Data Bank (www.rcsb.org), are depicted. The peptide chains are color-coded according to specific regions in the primary amino acid sequence: the N-terminus (hydrophilic or metal-binding region) from D1 to Q15 is colored beige; the central hydrophobic core (CHC) or β1 region from K16 to A21 is shown in cyan; the loop or central hydrophilic region from E22 to K28 is yellow; the second hydrophobic region (β2) from G29 to M35 is colored salmon; and the C-terminal region from V36 to A42 is green. The PDB codes are labeled above the corresponding structures. A possible mechanism for oligomer formation is the rapid assembly driven by hydrophobic interactions, including those involving the C-terminus [38].

The size of soluble oligomers ranges from 10 to 100 kDa and they exhibit significant heterogeneity and dynamic behavior. According to the widely recognized protein folding funnel model, the conformational space of a folding protein or peptide is vast [65,66,67,68,69,70]. Unfolded soluble monomers occupy the broad top of the funnel, possessing the highest energy and the ability to adopt numerous conformations. Folded intermediates or partially folded states, as well as oligomers and native forms of the monomer, are lower in energy and correspond to local minima within the energy landscape. Amorphous aggregates are located at one of the narrower bottoms of the funnel, characterized by deep energy minima, while the global free energy minimum, at the narrowest part of the funnel, is occupied by amyloid fibrils [66]. Fibrilization begins with an ensemble of unfolded monomer conformations and rapidly proceeds along various pathways toward the global minimum, where distinct amyloid polymorphs occupy closely positioned local minima. The absolute free energy minimum is associated with amyloid crystals [68,69]. The exact mechanisms and conformational transitions from disordered monomers through oligomers to fibrils remain challenging to understand, due to the heterogeneity, metastability, and dynamic nature of oligomers. Additionally, fibrillogenesis is strongly influenced by factors such as temperature, concentration, and the homogeneity of the starting monomer structures. Various Aβ oligomer structures, including U-shaped, S-shaped, LS-shaped, and those with two- or three-fold topologies, have been identified through solution and solid-state NMR and cryo-electron microscopy, with some originating from human sources, as shown in Figure 2 [71,72,73,74,75,76].

At the structural level, the predominant secondary structure of amyloid fibrils is the cross β-sheet, where the Aβ backbone is oriented orthogonally to the fibril axis [77,78,79,80,81]. In the oligomer mixture of the on-pathway, β-sheet structure is commonly observed. In solution, the Aβ monomer typically adopts an unfolded conformation rather than any distinct secondary structure (Figure 2). The primary structure of Aβ_1–42_ is divided into five regions: the N-terminus, also known as the hydrophilic or metal-binding region (D1 to Q15); the central hydrophobic core (CHC), spanning residues K16 to A21; the loop or central hydrophilic region (E22 to K28); the second hydrophobic region (G29 to M35); and the C-terminal region (V36 to A42) (Figure 2) [56].

## 4. Interactions Leading to and Stabilizing Aβ Aggregation

It has been found that within the β-hairpin structured monomers that form Aβ oligomers, intrastrand hydrogen bonds occur between the β-strand regions, specifically between I31 and V36 [82]. During the transformation from β-hairpin monomers to β-sheet secondary structure, these intrastrand bonds must be broken, and new interstrand hydrogen bonds are formed between adjacent peptide sequences [83]. This shift from intra- to interstrand hydrogen bonds is essential for the oligomerization process, resulting in the formation of the cross β-sheet structure. It has also been found that during the Aβ association process, monomers primarily interact with polar surfaces such as mica, while hydrophobic surfaces such as graphite disrupt the oligomer structure and act as templates for fibrillization [84]. Furthermore, it is thought that the initial interaction responsible for the β-sheet structure in mature fibrils involves a hydrophobic contact between F19 from the central hydrophobic core (CHC) of one peptide and L34 from the second hydrophobic region of another monomer [85,86,87]. A critical interaction that stabilizes the turn in the cross β-structure is the salt bridge between D23 and K28 [88,89,90]. During oligomerization, it is believed that monomer oligomerization occurs via parallel stacking in the axial direction of fibril extension. However, published fibril structures suggest that the dimeric Aβ_1–42_ unit, composed of two S-shaped monomers arranged in a C2 symmetric “ying-yang” manner, is involved in fibril growth [74,75,76]. It has been identified that the key interactions stabilizing all types of quaternary fibril structures involve the side chains of M35 and one or more residues, such as I31, I32, and M35 from one monomer, interacting with G37, G39, and V29 from a second monomer [91,92,93,94,95]. In the U-shaped Aβ_17–42_ form, K28-D23 salt bridges serve as key stabilizing interactions [96]. In contrast, the S-shaped Aβ_11–42_ and LS-shaped Aβ_1–42_ fibrils are stabilized by salt bridges between the positively charged NH^3+^ group of the K28 side chain and the negatively charged COO^−^ group of A42 [73,75]. Additionally, in the LS-shaped form, the N-terminal and C-terminal regions are reinforced by E11-H6/H13 hydrogen bonds, which play a crucial role in fibril stability. Recent studies emphasize the importance of the hydrophobic core formed by F4, L34, and V36, along with the K28-A42 salt bridge, in stabilizing the LS-shaped Aβ fibril [75].

## 5. Molecular Dynamics Simulations: Challenges and Prospects

Understanding the mechanisms by which small organic molecules inhibit oligomerization and designing new inhibitors is a complex challenge for traditional experimental techniques. This difficulty arises from the vast conformational space occupied by Aβ monomers and oligomers, the metastable nature of oligomers during fibrillization, and their dynamic structural changes. Computational approaches, such as molecular dynamics simulations, serve as valuable complementary tools in this research field.

Molecular dynamics (MD) is a powerful structure-based drug design (SBDD) method that enhances our understanding of the properties, functions, and mechanisms of action or inhibition of biomacromolecules and other systems of interest. MD is currently the only applicable method capable of simulating the internal motions of biomolecules at the atomic level, providing insights that are otherwise unattainable using conventional experimental techniques [97,98]. Integrating experimental data with MD simulations leads to a more comprehensive understanding of molecular processes and mechanisms. In conventional or classical MD (cMD), also known as all-atom MD, each atom is represented as a particle that has position and velocity, evolving according to Newton’s laws of motion. The interactions between atoms are calculated using force fields (FFs), which are mathematical models that determine the potential energy of a system by accounting for bonded and non-bonded interactions. These parameters can be derived from experimental data or computed using quantum mechanics. Since different molecular systems, such as proteins, nucleotides, and small organic molecules, require specialized force fields, no single universal FF applies to all cases. Well-established force fields for proteins include Amber ff14S [99], GROMOS versions 54A7 and 54B7 [100], CHARMM [101], and OPLS3 [102]. Most of these force fields are optimized for structured proteins. However, since Aβ peptides belong to the category of intrinsically disordered proteins (IDPs), specialized FFs have been developed to describe their dynamic behavior more accurately. A recent review by Boopathi and colleagues extensively discusses various force fields tailored for IDPs [56]. Some of these include ff99IDPs, ff14IDPSFF [103], ff14IDPs [104], ff03 CMAP [105], ff14SB [99], CHARMM36m [106], and CHARMM36IDPSFF [107], all designed for better modeling of misfolded and folded protein structures [108,109,110,111,112,113].

While specialized force fields have been developed for IDPs, their suitability depends on system design and simulation conditions, such as sampling methods and water models. Furthermore, comparisons between different FFs are based on diverse criteria, as shown in Table 1. Various software packages also recommend specific FFs and water models for IDPs. For instance, the latest version of Amber24 suggests using the ff99SD force field with the general-purpose four-point OPC water model [114,115].

Carballo-Pacheco and Strodel highlighted that the error in calculating NMR observables is so significant that determining which force field best represents experimental data remains challenging [116]. Additionally, multiple studies present conflicting results regarding the most accurate force field for modeling IDPs and protein aggregation. However, newer force fields generally outperform older ones, reflecting continuous advancements in their accuracy over time.

**Table 1:** Comparison of force fields for IDPs based on various criteria and system specifications. Force fields are marked in bold if the authors conclude that their findings align with experimental data.

Examined FFs	Criteria for Comparison	Studied System/s	Sampling Method/ Water Model	Ref.
AMBER94, AMBER96, AMBER99, **AMBER99-ILDN,** AMBER03, AMBER12SB, **AMBER14SB,** GROMOS43a1, GROMOS43a2GROMOS45a3, GROMOS53a5, GROMOS53a6, GROMOS54a7, CHARM22*, CHARRMM36, CHARRMM36m, and OPLS-AA	global reaction coordinates, secondary structure content, fibril population and formation	100 dimeric Aβ_16–22_	cMD/TIP3P for all FFs, except for GROMOS FFs―SPC, CHARM22*―mTIP3P, and OPLS―TIP4P	[117]
ff99SB, **ff14SB,** FF14SB_IDPs, CHARMM36, **CHARMM36m**	chemical shifts, secondary structure content, nonbonded energy component, E_nonbonded_	monomeric Aβ_42_	cMD and REMD/TIP3P	[118]
ff99IDPs, ff14IDPs, ff14IDPSFF, ff03w, CHARMM36m, and CHARMM22*	chemical shits, J-couplings, global reaction coordinates, secondary structure content	RS-peptide, HEWL19, HIV-rev, Aβ_40_, Aβ_42_, phosphodiesterase-γ, CspTm, and ubiquitin	cMD/for ff99IDPs, ff14IDPs, ff14IDPSFF―TIP3P, for ff03w―TIP4P-2005; and for CHARMM FFs―CHARMM-modified TIP3P	[113]
Gromos54a7, OPLS-AA, AMBER03ws, CHARMM22*, and AMBER99SB*ILDN	oligomer formation kinetics, in terms of dissociation constant, K_D_ and ∆G, and collision acceptance probability	monomeric forms and six monomers of each of the three peptides: Aβ_16–22_, one non-amyloidogenic mutant (F19V, F20V), and the Aβ_16–22_ (F19L) mutant, which exhibits rapid fibrillogenesis	SPC for Gromos54a7, TIP4P/2005 for AMBER03ws, TIP4P for OPLS-AA, TIP4P-Ew for CHARMM22* and AMBER99SB*ILDN	[119]
OPLS, AMBER99SB, AMBER99SB*ILDN, AMBER99SBILDN-NMR and CHARMM22*	local NMR observables, including chemical shifts, J-couplings, and residual dipolar couplings (RDCs)	monomeric Aβ_1–42_	REMD/TIP4P-Ew for AMBER99SB, AMBER99SB*ILDN, AM-BER99SBILDN-NMR and CHARMM22*, TIP3P for OPLS	[116]

Abbreviation: REMD―replica exchange molecular dynamics.

The derivatives of the potential energy functions correspond to the forces in Newton’s equations of motion, which are solved iteratively for each atom in time steps of femtoseconds. While cMD enables highly precise calculations, its simulations are limited in timescale, typically reaching only microseconds to milliseconds, even with modern computational advancements. However, given the stochastic nature of biological processes, this duration is often insufficient to achieve comprehensive Boltzmann sampling or to capture critical changes in biologically relevant phenomena such as protein folding and amyloid formation. To overcome these drawbacks, including timescale limitations and sampling deficiencies, various advanced sampling methods have been developed using different techniques [120,121,122,123,124,125,126]. A comprehensive review and classification of the numerous sampling methods was published by Zuckerman [124]. Here, we will briefly describe some of them, considering the scope and purpose of this review. In this review, we will briefly highlight some of these methods, focusing on the scope and objectives of our discussion. For in-depth discussions on cMD and advanced MD techniques, refer to several articles [121,124,125,126,127,128,129,130,131,132].

The generalized-ensemble algorithm enables a system to bypass energy barriers of local minima and sample a much broader conformational space [133,134,135]. This is achieved by replacing the Boltzmann probability weight factor, which decreases exponentially with the energy barrier height, with non-Boltzmann factors, ensuring a uniform distribution of potential energy space. As a result, a random walk in temperature leads to a random walk in potential energy. One example of this approach is simulated tempering (ST), which requires the initial determination of probability weight factors, a computationally demanding task [136,137]. This issue is bypassed in the widely used replica exchange method (REM) [138,139,140,141,142] (also known as parallel tempering [133,140,142,143], replica Monte Carlo [90,91,138], and multiple Markov chain method [139]), where the probability weight factor is predefined. The method involves simulating multiple non-interacting replicas independently, each representing a copy of the original system in the canonical ensemble at different fixed temperatures. Periodically, replicas at neighboring temperatures are swapped based on the Metropolis criterion. The replica exchange method applied to MD, called REMD (replica exchange molecular dynamics), was developed by Sugita and Okamoto [144]. In REMD, in addition to considering coordinates and potential energy functions, momenta are accounted for, and the velocities of all atoms in a pair of replicas are uniformly rescaled. This method is particularly suitable for parallel computing, as each temperature exchange must be synchronized. Therefore, the application of this method to complex biological systems with explicit solvent molecules is constrained by computational power, as these systems have an immense number of degrees of freedom, necessitating the simulation of a large number of replicas simultaneously on a vast, homogeneous computer cluster. To address this challenge, various variants and optimizations of the replica exchange method have been developed [145,146,147,148,149,150,151,152,153,154,155,156].

A relatively recent method based on the generalized-ensemble algorithm is simulated tempering distributed replica (STDR), which is designed for use on distributed or heterogeneous platforms [130,157]. The method requires minimal initial simulation, unlike ST, and is not affected by replica synchronization. Additionally, it does not require a fixed number of temperatures, unlike RE. However, when using generalized-ensemble methods, it is important to consider the potential loss of dynamic information typical of cMD, due to non-physical transitions and changes that may occur during simulations.

One approach to overcome the limitations of cMD is to reduce the degrees of freedom. It is clear that increasing the system size raises the degrees of freedom, which, in turn, limits the duration of simulations. Coarse-grained (CG) modeling, where a single particle represents a group of atoms instead of individual atoms, significantly reduces the degrees of freedom, allowing simulation timescales to be extended by 2–4-times compared to atomistic representations, though this comes at the cost of reduced accuracy. Various coarse-grained models (CGMs) exist, ranging from one-bead to united-atom representations [158,159,160]. For example, a coarse-grained model for proteins is implemented in the MARTINI force field [161]. Another method for reducing degrees of freedom is by representing the solvent implicitly using implicit solvent-based approaches [162].

An advanced strategy for improving conformational sampling and overcoming the timescale limitations of cMD involves modifying the potential energy in the force field. Techniques like conformational flooding involve incorporating a flooding potential into the force field, which destabilizes the local energy minima, facilitating the escape from initial energy wells without altering the reaction pathway [163,164]. Another method is metadynamics, also known as the local elevation method. This technique involves adding a Gaussian-type biasing potential to the Hamiltonian, pushing the system away from previously visited conformational states [165,166,167,168,169]. It requires prior selection of significant coordinates to which repulsive terms are applied. Metadynamics is particularly effective for systems with a smooth energy landscape. Another technique is umbrella sampling, where an artificial biasing potential, referred to as the umbrella, is incorporated into the potential energy function. This bias directs the sampling towards less favorable conformational regions. The umbrella potential acts as a compensating function, typically concentrating the sampling within a specific region, known as a window. By shifting the focus through multiple simulations, the entire conformational space can be explored. Prior knowledge of the reaction coordinate is essential for constructing the umbrella potential, as the method heavily relies on the chosen potential and the number of windows [170,171]. In accelerated MD (aMD), the potential energy landscape of the original system is modified by introducing a bias potential, which raises the energy minima of the wells and reduces the energy barriers between different states. The advantage of this approach is that it does not require prior knowledge of the system’s potential energy landscape, eliminating the need for defining the reaction coordinate in advance. Initially introduced by Voter under the name “hyperdynamics,” the goal was to shorten the residence time of the system within the potential minima and overcome energy barriers, thereby enabling the study of long-term system behavior [172,173]. The early version of this bias potential was time-consuming and limited to small systems. Later, Hamelberg and colleagues developed a bias potential that could be applied to larger systems, such as biomolecules [174]. The key innovation of this bias potential is that it preserves the overall shape of the system’s potential energy surface. To ensure that thermodynamic quantities, like free energy changes derived from the modified potential, match those from the unmodified potential, a reweighting step is necessary. However, in systems with large biases, typical of large systems, the exponential reweighting factor causes the ensemble averages to rely on only a few configurations with high weights, reducing the data points and affecting the accuracy of thermodynamic quantities [175]. To address this issue, variants of aMD have been developed, including replica exchange accelerated molecular dynamics (REXAMD) [176], selective aMD [177], and others [178,179,180].

Despite the development of numerous advanced techniques, and while they are often highly effective, it is important to remember that there is no single universal method that can serve as an ideal solution for the timescale limitations of cMD in all cases [181,182].

Alongside the development of MD sampling methods, force fields and algorithms have been continuously improved (some examples can be found [183,184,185]). Additionally, computational power continues to expand. These advancements have contributed to extending the timescale of simulations, making MD simulations more accessible and indispensable for uncovering the functions and mechanisms of biological systems. For example, the latest version of the supercomputer designed specifically for conventional MD simulations of biological systems, Anton 3, can perform cMD simulations of systems with a million atoms at over 100 microseconds per day [186]. The third-fastest supercomputer on the Top-500 June 2023 list [187], LUMI (Rmax (PFlop/s) 309.10), can simulate 80 nanoseconds per day for a system with over a million atoms [188]. The second-fastest supercomputer on the same list is capable of simulating 8.30 ns per day for a system of 1.6 billion atoms, allowing for cellular-scale simulations [184]. In addition to the evolution of supercomputers, the distributed computing project Folding@home (FAH or F@h) was developed [189,190]. In 2020, during the pandemic, over a million citizen scientists contributed their computing resources to simulate the SARS-CoV-2 proteome, making the FAH platform one of the largest computational resources in the world. It was the first time it crossed the exascale computing barrier and successfully simulated 0.1 s of the viral proteome [191].

In 2012, Lemkul and Bevan reviewed the pioneering in silico studies on amyloid beta inhibitors, including molecular docking and molecular dynamics simulations of both Aβ fragments and the full-length peptide [192]. They also highlighted the limitations of existing methods used for Aβ systems and fibrils, such as the restricted timescales of production dynamics, the use of implicit solvent models, and the constraints of scoring functions.

Over the past decade, only a few articles have reviewed the applications of SBDD and methods, including molecular docking and/or molecular dynamics simulations, for various Aβ inhibitors [56,193,194,195]. To our knowledge, none have focused on the mechanistic insights gained from MD simulations of endogenous compounds and repurposed drugs, which is the main objective of this review.

Endogenous compounds—naturally synthesized within the human body—present a promising approach to disease treatment. This strategy is considered one of the most natural, leading to minimal or no toxicity and being well tolerated with few or no side effects. These compounds have demonstrated superior therapeutic outcomes. Another effective approach is drug repurposing, discovering new therapeutic applications for existing drugs.

The following sections focus on MD studies that uncover the mechanisms of these two classes of small molecules. Given the limited research over the past decade specifically targeting the toxic Aβ_1–42_ form, we also include MD studies on the Aβ_1–40_ isoform of the peptide.

## 6. Endogenous Compounds Inhibiting Aβ

### 6.1. Dopamine (DA) and Norepinephrine (NE)

**Figure 3:**
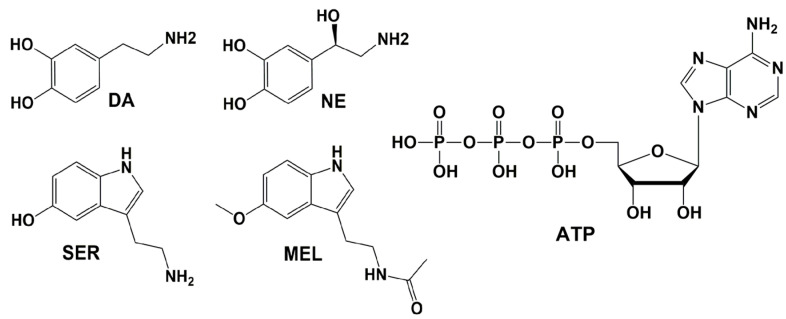
Depiction of the endogenous compounds studied through MD simulations.

Dopamine (DA) and norepinephrine (NE) (also known as noradrenaline) (Figure 3) belong to the catecholamine family and function as neurotransmitters and neuromodulators. In the brain, DA is involved in motor control, executive functions, and the motivational aspects of reward-driven behavior [196]. NE mobilizes the brain and body for action, playing a vital role in maintaining alertness, attention, mood, memory, and stress response [197]. Additionally, it is crucial for supporting contextual and spatial memory [198].

A study of the anti-amyloidogenic and fibril-destabilizing effects of anti-Parkinsonian agents found that DA dose-dependently inhibits the formation and extension of Aβ_1–40_ and Aβ_1–42_ fibrils, as well destabilizing preformed fibrils [199]. Another study revealed that catechol derivatives, including catechol, DA, L-DOPA, NE, epinephrine, and L-tyrosine, primarily inhibit nucleation rather than the elongation of Aβ fibrilization in the presence and absence of a model biomembrane [200]. Among the studied compounds, DA exhibited the strongest inhibitory activity, followed by L-DOPA and NE, in preventing Aβ_1–40_ and Aβ_1–42_ fibril formation.

A REMD study on Aβ_1–40_ protofibril (Table 2) revealed that DA preferably binds to β-2 BS, a β-sheet of IIGLMVG (residues 31–37) from the second hydrophobic region of Aβ, as well as to the N-terminal site, which presents disordered tails [201]. The study found that the ligand significantly impacted the oligomer’s double-layered structure.

In a comprehensive study conducted by Chen et al., the disruptive mechanisms of DA were investigated using cMD simulations of protonated DA^+^ molecules at different molecular ratios with preformed pentameric Aβ protofibrils, as well as REMD simulations of DA^+^ with Aβ dimers (Table 2) [202]. The authors found that in the systems with low molar ratios (1:1 and 2:1), DA^+^ molecules destabilized Aβ protofibrils in a dose-dependent manner by inserting into the F4-L34-V36 core region and disrupting intra- and interchain K28-A42 salt bridges. In the 1:1 system, DA^+^ molecules primarily bound to the turn-1 region (H6-H13), while those in the 2:1 system also interacted with the F4-L34-V36 core region, the N-terminal (D1-R5), the turn-2 region (F20-D23), and the C-terminal (I41 and A42). These interactions occurred through hydrogen bonding with residues D1, E3, H6, D7, E11, H13, Q15, E22, D23 and A42; salt bridge formation with D7 and E11 (for the 1:1 system) and E11, E22 and D23 (for the 2:1 system); cation-π interactions with R5; and π-π stacking with F4, H6, H13, H14, F19 and F20.

At higher concentrations (molar ratio 10:1), the protofibril structure remained intact as the DA^+^ molecules predominantly bound to the outer surface of Aβ protofibril, limiting its flexibility (Table 2). However, when 10 DA^+^ molecules were replaced with deprotonated DA^0^—which exists at low concentrations under physiological pH—DA^0^ molecules preferentially bound to the inner surface of the protofibril, particularly within the F4-L34-V36 hydrophobic core. The deprotonated DA^0^ molecules interacted with protonated DA^+^ molecules via π-π stacking, enhancing their binding to the inner surface and ultimately exerting a disruptive effect on the protofibril structure. Additionally, the authors examined the inhibitory effects of DA^+^ molecules on Aβ dimerization (Table 2) [202].

The mechanism of Aβ dimer aggregation inhibition and fibril destabilization by NE was studied using REMD and cMD simulations, respectively (Table 2) [203]. During dimer fibrillization, NE was found to decrease β-sheet content while increasing α-helix, coil, and turn content. Five main binding sites (BSs) were identified: (1) 3EFRHD7, (2) 10YEVHH14, (3) 16KLVFFA21, (4) 31IIGLMV36, and (5) 39VVIA42. Among these, the third and fourth sites were the most favorable, as they had the lowest binding energy. NE molecules primarily interacted with hydrophobic residues I41, I31, and L17, as well as aromatic residues Y10, F4, and F20, through hydrophobic and stacking interactions, respectively. Additionally, hydrogen bonds and cation-π interactions contributed to NE binding to Aβ peptides. Hydrogen bonds were mainly formed with negatively charged aspartic and glutamic acid residues (D1, E3, D7, E11, E22, and D23), while cation-π interactions occurred with the positively charged side chain of R5.

**Table 2:** Summary of the technical data and key conclusions from the reviewed MD simulations. Most studies applied conventional MD, while some utilized alternative methods, which are specified accordingly.

FF/Water Model	Duration per System, ns	Aβ Length/PDB ID/Type (Monomer/Dimer/ (Proto-)Fibril)	Inhibitor *	Main Findings	Ref.
REMD/GROMOS 57a7/SPC	50 per replica	Aβ_1–40_/2LMN/decamer, protofibril	DA	preferably binds to β-2 and N-terminal; significantly affects the oligomer’s double-layer structure	[201]
AMBER99SBILDN/TIP3P	5 × 500 per system	Aβ_1–42_/5OQV/pentamer	(1) 5 DA^+^* (molecular ratio 1:1) (2) 10 DA^+^ (2:1) (3) 50 DA^+^ (10:1) (4) 40 DA^+^ + 10 DA^0^ (10:1)	(1) binds to H6-E11 (turn-1 region); weak disruptive effect; (2) binds to H6-E11, the F4-L34-V36 hydrophobic core, the turn-2 region (F20, E22 and D23), and the C-terminal residues I41 and A42; stronger disruptive effect (3) binds mainly to the outer surface, decreasing the flexibility of the Aβ protofibril and stabilizing it (4) DA^0^ molecules bind to the inner surface of the protofibril, primarily to the F4-L34-V36 hydrophobic core; π-π stacking with DA^+^ increases their inner surface binding, resulting in a disruptive effect of the DA^0^ and DA^+^ mixture	[202]
REMD/AMBER99SBILDN/TIP3P	950 per replica (48 replicas)	(1) Aβ_1–42_/1IYT/two monomers placed in three orientations: parallel, antiparallel and perpendicular	10 DA^+^ (molar ratio 5:1)	inhibits the dimerization	[202]
REMD/AMBER99SBILDN/TIP3P	300 per replica (54 replicas)	Aβ_1–42_/1Z0Q/dimer	20 NE (molar ratio 10:1)	suppresses and reduces the interpeptide β-sheet content; five dominant BS were identified; main contact residues: hydrophobic interactions with L17, I31, and I41; π-π stacking with Y4, F10, and F20; H-bonds with D1, E3, D7, E11, E22, and D23; cation-π interactions with R5	[203]
AMBER99SBILDN/TIP3P	2 × 1000 (2 × 1 µs)	Aβ_1–42_/5OQV/pentamer	100 NE	β-sheet content decreased, while coil content increased; the number of fibril H-bonds decreased; H-bonds formed with D1, A2, D23, and A42; destabilizes the preformed fibril	[203]
AMBER99SBILDN/TIP3P	3 × 500 (per system)	Aβ_1–42_/5OQV/tetramer	(1) 20 NE^+^ (molar ration 5:1) (2) 20 NE^0^ (molar ration 5:1)	(1) destabilizes the protofibril; primarily disrupts the D1-H14 region; decreases β-sheet content; increases the kink angle around Y10; disrupts H6-E11 H-bonds and K28-A42 salt bridges; forms H-bonds with E3, D7, E11, Q15, E22, and D23; engages in π-π stacking with H6, H13, and F20; (2) disrupts the protofibril; decreases β-sheet content in dispersed regions, including A2–H6, A21–G22, S26, and M35–V36; primarily forms hydrophobic interactions and π-π stacking with F4, R5, H6, Y10, H13, Q15, F20 and L34.	[204]
AMBER99SBILDN/TIP3P	3 × 500	Aβ_1–42_/5OQV/tetramer	40 SER (molar ratio 10:1)	disrupts fibrils by decreasing β-sheet content at the N-terminal (D1-Y10); main contact residues include F4, H6, Y10, H13, Q15, and L34; dominant interactions involve π-π stacking with F4, H6, Y10, and H13; disrupts fibril-stabilizing contacts A2-V36 and F4-L34 at core-1; as well as salt bridges between L34-A42.	[205]
AMBER99SBILDN/TIP3P	3 × 500	Aβ_1–42_/5OQV/tetramer	40 MEL (molar ratio 10:1)	disrupts fibrils by decreasing β-sheet content at the N-terminal (D1–Y10) and C-terminal (D23–A42); two BSs were identified: (1) at the N-terminal (F4, H6, Y10, H13, H14, Q15, L17, and F19) and (2) at the C-terminal (N27, I31, I32, L34, and V36); dominant interactions include π-π stacking with F4, H6, Y10, H13, H14, and F19, as well as hydrophobic interactions with N27, I31, I32, L34 and V36; disrupts fibril-stabilizing contacts A2-V36 and F4-L34 at core-1, L17-I31 at core-2, and I32-M35 at core-3, along with salt bridges between L34 andA42	[205]
AMBER14SB/TIP3P	(1) 500 (2) 500 (3) 3 × 500	Aβ_16–22_/random pentamer	(1) 20 ATP (molar ratio 4:1) (2) 25 ATP (molar ratio 5:1) (3) 30 ATP (molar ratio 6:1)	with increasing ATP concentration, (i) β-sheet content decreased, while turn, bend, and coil contents increased, preventing oligomerization; (ii) the ATP-F π-π stacking disrupted the F-F interpeptide interactions; (iii) peptide-peptide H-bonds decreased, while ATP-peptide H-bonds increased;	[206]
AMBER99SB-ILDN/TIP3P AMBER-FB15/TIP3P-FB	2 × 500 (for each FF)	Aβ_16–22_/random pentamer	30 ATP (molar ratio 6:1)	β-sheet content decreases sharply; interpeptide F-F interactions are reduced; peptide-peptide H-bonds decrease abruptly, preventing β-sheets formation;	[206]
AMBER14SB/TIP3P AMBER99SB-ILDN/TIP3P AMBER-FB15/TIP3P-FB	500 (for each FF)	Aβ_16–22_/dimer	16 ATP (molar ratio 8:1)	unfavored dimerization	[206]
AMBER14SB/TIP3P AMBER99SB-ILDN/TIP3P AMBER-FB15/TIP3P-FB	500	Aβ_16–22_/prefibrillar pentamer	150 ATP (molecular ration 30:1)	destabilizes β-sheet content and promotes disaggregation; completely disaggregates fibrils in the AMBER14SB and AMBER-FB15 FFs; a significant decreases inter-peptide H-bonds	[206]

DA—dopamine; NE—norepinephrine; SER—serotonin; MEL—melatonin; ATP—adenosine triphosphate; BS—binding site; * number of molecules is specified when different from 1; ^+^—protonated form; ^0^—unprotonated form.

In the fibril system, NE molecules led to a decrease in β-sheet content while increasing coil content [203]. NE molecules remodeled the Aβ fibril structure through hydrogen bonding with D1, A2, D23, and A42 residues, ultimately destabilizing the preformed fibril.

cMD simulations of the protonated (NE^+^) and deprotonated (NE^0^) forms of norepinephrine revealed that both forms disrupt the LS-shaped tetrameric protofibril through distinct mechanisms (Table 2) [204]. The protonated form, which is the predominant form at physiological pH, destabilized the entire protofibril by primarily disrupting the N-terminal region (D1–H14), reducing its β-sheet content, and increasing the kink angle around Y10. It formed hydrogen bonds with E3, D7, E11, Q15, E22, and D23, as well as π-π stacking interactions with H6, H13, and F20. As a result, the H-bonds between H6 and E11 along with the salt bridges between K28 and A42—both critical for fibril stabilization—were disrupted. The deprotonated form disrupted the protofibril by reducing β-sheet content in several scattered regions, including A2–H6, A21–E22, S26, and M35–V36. Its interactions were primarily hydrophobic and involved π-π stacking with F4, H6, Y10, H13, Q15, F20, and L34. The interactions with F4 and L34 likely interfered with the essential stabilizing contacts between these residues, ultimately disrupting the F4–L34–V36 hydrophobic core.

### 6.2. Serotonin (SER) and Melatonin (MEL)

Serotonin (SER) (Figure 3), an indoleamine neurotransmitter involved in regulating mood, cognition, learning, and memory, and its derivative melatonin (MEL), a hormone essential for controlling the circadian rhythm or sleep–wake cycle, are both synthesized in the brain from the amino acid tryptophan. In patients with Alzheimer’s disease (AD), cortical serotonin levels are reduced due to acute tryptophan depletion, contributing to cognitive decline [207,208]. Additionally, SER exhibits neuroprotective effects against Aβ toxicity and effectively inhibits Aβ fibrillization [209,210]. MEL levels decline with age, with a more pronounced reduction observed in AD patients [211,212,213,214]. MEL has been shown to exert a neuroprotective effect by inhibiting Aβ aggregation; it directly interacts with Aβ peptides, prevents amyloid beta formation, and promotes Aβ fibril dissociation [215,216,217,218].

A recent study by Gong et al. investigated the molecular-level effects of SER and MEL on LS-shaped Aβ fibrils (Table 2) [205]. Interestingly, both indoleamine derivatives exhibited distinct mechanisms of Aβ fibril destabilization. SER primarily binds to the N-terminal region (D1-Y10), disrupting its β-sheet content mainly through π-π stacking interactions with F4, H6, Y10, and H13. As a result, it interferes with the fibril-stabilizing interactions between A2 and V36, as well as between F4 and L34. The binding site (BS) residues of SER include F4, H6, Y10, H13, Q15, and L34, as SER mainly interacts with inner-surface-oriented residues whose side chains are embedded within the Aβ protofibril, such as F4, H6, H13, Q15, and L34. Furthermore, SER insertion induces distortion of the C-terminal region (mainly D23-A42) and disrupts intra- and interchain K28–A42 salt bridges, with a stronger effect on intrachain interactions.

In contrast, MEL binds to two binding sites on the L-shaped Aβ protofibril—one at the N-terminal segment, comprising residues F4, H6, Y10, H13, H14, Q15, L17, and F19, and the other at the C-terminal region, including residues N27, I31, I32, L34, and V36. MEL disrupts the β-sheet structure in both the N-terminal (D1-Y10) and C-terminal (D23-A42) segments, exhibiting a stronger destabilizing effect on the entire protofibril. It interferes with fibril-stabilizing interactions at three hydrophobic cores: A2-V36 and F4-L34 at core-1, L17-I31 at core-2, and I32-M35 at core-3. The impact of MEL on L34-A42 inter- and intrachain salt bridges is more significant than that of SER, with a greater effect on intrachain interactions. MEL primarily engages in two types of intermolecular interactions: π-π stacking with aromatic amino acids in the N-terminal region, such as F4, H6, Y10, H13, H14, and F19, and hydrophobic contacts with C-terminal residues, including N27, I31, I32, L34, and V36.

### 6.3. Adenosine Triphosphate (ATP)

Adenosine triphosphate (ATP) (Figure 3) is recognized as the primary energy source for various processes in living cells. Experimental studies have shown that ATP acts as a biological hydrotrope—an amphiphilic molecule that, unlike conventional surfactants, exhibits low aggregation cooperativity and functions at molar concentrations. ATP prevents the formation of peptide aggregates and dissolves pre-existing ones at the millimolar scale [219]. Recently, an experimental study revealed that ATP induced amorphous aggregation of Aβ by increasing its dynamics [220].

Coskuner and Murray studied the effect of ATP on Aβ fibrils, both computationally and in vitro, finding that ATP reduced Aβ misfolding at physiological intracellular concentrations of approximately 500 µM [221]. The in silico calculations, which included initial molecular docking followed by short 10 ns cMD simulations on the Aβ_9–40_ fibril and thermodynamic analyses, showed that ATP primarily interacted with Y10 and S26, with Y10 playing a crucial role in ATP binding to the fibril.

A thorough investigation by Pal and Paul examined the inhibitory effect of ATP on the central hydrophobic core (CHC) Aβ_16–22_ of the Aβ peptide using three different force fields: AMBER14SB, AMBER99SB-ILDN, and AMBER-FB15. The peptide was considered as a dimer, random pentamer, and prefibrillar pentamer at different molecular ratios (Table 2) [206]. Trajectory analyses of the random pentamer simulations across the three FFs showed similar results in the presence of ATP. Specifically, ATP inhibited the oligomerization of Aβ peptides at millimolar concentrations, as indicated by a decrease in β-sheet content, peptide–peptide hydrogen bonds, and intrapeptide F-F interactions. Meanwhile, ATP-F π-π stacking interactions and ATP-peptide hydrogen bonds increased. The MD simulations of the dimer systems across all three FFs showed that ATP inhibited dimerization. Additionally, ATP destabilized the β-sheet content and induced disaggregation in pre-formed fibril systems simulated with AMBER14SB, AMBER99SB-ILDN, and AMBER-FB15 FFs. Notably, in the AMBER14SB and AMBER-FB15 FFs, the fibrils were completely disaggregated. These results were supported by a significant decrease in interpeptide H-bonds.

## 7. Repurposed Drugs Inhibiting Aβ

### 7.1. Propafenone (PPF)

**Figure 4:**
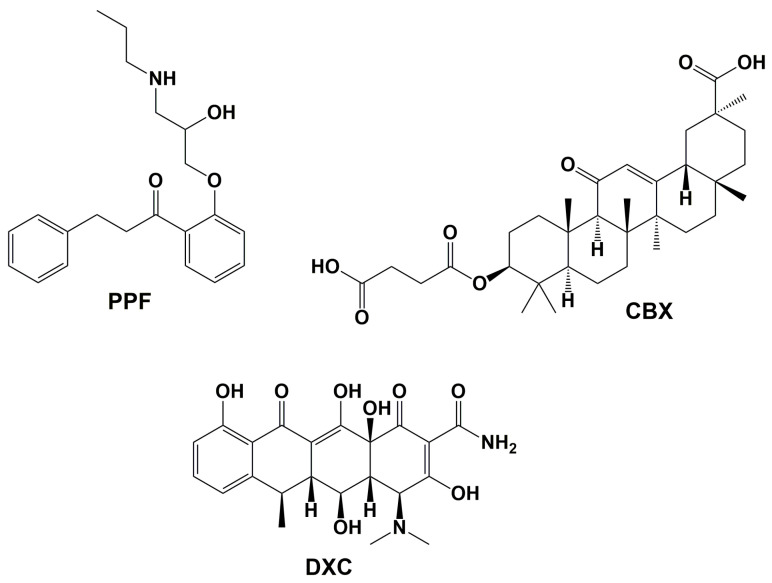
The structures of the repurposed drugs (Propafenone (PPF), Carbenoxolone (CBX), and Doxycycline (DXC) studied through MD simulations are presented.

To discover novel anti-amyloidogenic agents, one approach involves repurposing existing approved drugs, while another strategy focuses on screening compounds with high similarity to known inhibitors. Combining these methods, Ngo et al. conducted a similarity search of FDA-approved drugs with over 80% similarity to curcumin, followed by molecular docking against the Aβ_9–40_ fibril, a dodecamer with two-fold symmetry [222]. They then performed cMD simulations (Table 3) of the selected drugs, followed by absolute binding energy calculations, which identified Propafenone (PPF) (Figure 4), an anti-arrhythmic drug, as the most promising Aβ inhibitor. Further trajectory analyses revealed that PPF was located near the turn in the lower layer of the dodecameric fibril, primarily interacting with the hydrophobic residues. Additionally, it was found that β-content decreased in the presence of PPF, potentially leading to fibril degradation. Experimental validation further confirmed the anti-amyloidogenic activity of PPF against Aβ_40_ (IC_50_(_PPF_) = 1.8 µM, IC_50_ (_curcumin_) = 2.7 µM) and Aβ_42_ (IC_50_(_PPF_) = 3.9 µM, IC_50_ (_curcumin_) = 6.9 µM), as well as its antioxidant properties and ability to reduce Aβ_40_-induced cytotoxicity. PPF also exhibited protective effects against Aβ_40_- and Aβ_42_-induced toxicity in SH-SY5Y cells.

### 7.2. Carbenoxolone (CBX)

Carbenoxolone (3-*O*-(3-Carboxypropanoyl)-3β-hydroxy-11-oxoolean-12-en-30-oic acid) (Figure 4) is a natural derivative of an 18-glycyrrhetinic acid with a steroid-like structure found in liquorice root. It is a drug with pleotropic pharmacologic activity [223] and has been clinically used to treat various types of ulceration and inflammation. Known as a moderately potent gap junction blocker [224], the compound is water-soluble and can cross the blood–brain barrier (BBB). Carbenoxolone has shown nootropic and neuroprotective effects [225,226]. It reversibly inhibits the activity of 11β-hydroxysteroid dehydrogenase type 1 (11β-HSD1), which catalyzes the conversion of inactive cortisone to the active glucocorticoid cortisol in the brain, resulting in memory improvement [227]. Sharma et al. studied the inhibitory effect of CBX on Aβ aggregation, both experimentally and computationally [228]. The authors initially predicted binding sites on the monomeric and fibrillar forms of Aβ_1–42_ using the CASTp and RaptorX servers, followed by molecular docking calculations. In the final step of their computational analysis, they ran classical MD simulations of the best-docked complexes between CBX and both forms of Aβ_1–42_ forms for 10 ns (Table 3). They found that CBX reduced the secondary structure of both Aβ_1–42_ forms, decreasing α-helix and β-sheet content in the monomeric form and β-sheet content in the fibrillar form, while increasing unstructured content. The ligand formed contacts with F4, R5, H6, Y10, V12, H14, Q15, K16 V18, F19, and A30 in the monomer and with L17, V18, F19, F20, A21, D23, K38, L34, V36, V40, and I32 in the fibril chains. The compound also formed hydrogen bonds with R5, Q15, and F4 in the monomer and with F19 and D23 in the fibril, disrupting the salt bridge between D23 and K38.

### 7.3. Doxycycline (DXC)

In vivo and in vitro studies of the anticancer agent Iododoxorubicin have indicated its anti-amyloidogenic properties [229,230,231,232]. However, due to its significant toxicity, particularly cardiotoxicity, efforts have focused on identifying structurally similar compounds that retain anti-fibrillogenic activity while exhibiting a safer toxicological profile and have already been tested in clinical settings. Tetracyclines, which contain a polycyclic conjugated moiety similar to that of iododoxorubicin, have been shown to exhibit anti-amyloidogenic properties against different proteins, including Aβ [233,234,235,236,237,238,239,240,241,242,243]. Doxycycline (DXC) has been found to inhibit Aβ_42_ aggregation and disassemble mature amyloid fibrils [235,239,240].

**Table 3:** Repurposed drugs. A summary of the technical data and key findings from the reviewed MD simulations. Conventional MD was used in most studies, with alternative methods indicated in some cases.

FF/Water Model	Duration per System, ns	Aβ Length/PDB ID/Type (Monomer/Dimer/ (Proto-)Fibril)	Inhibitor	Main Findings	Ref.
GROMOS96 43a1/SPC	4 × 25	Aβ_9–40_/2LMN/dodecamer Aβ_1–40_/2M4J/nonamer	PPF	Mainly forms contacts with hydrophobic residues; β-content decreases;	[222]
GROMOS96/SPC	10	(1) Aβ_1–42_/1IYT/monomer (2)Aβ_17–42_/2BEG/pentamer, protofibril	CBX	(1) reduces α-helix and β-sheet secondary structure; increases unstructured contend; forms contacts with F4, R5, H6, Y10, V12, H14, Q15, K16 V18, F19, and A30; forms H-bonds with R5, Q15, and F4; (2) reduces β-sheet secondary structure; increases unstructured content; forms contacts with L17, V18, F19, F20, A21, D23, K38, L34, V36, V40, and I32; forms H-bonds with F19 and D23; disrupts the salt bridge between D23 and K38.	[228]
aMD/ff14SB/TIP3P	3 × 1000 (1 µs)	(1) Aβ1_1–42_/2MXU/pentamer (2) Aβ_1–42_/5OQV/pentamer	5 DXC (molar ratio 1:1)	(1) destabilizes the hydrophobic core (N15-A30); three main BSs: (i) near the M35 side chain; (ii) between I32 and L34; and (iii) between L17 and F19; (2) two BSs were identified: (i) near E1, V39, and I41; and (ii) at K16, V18, and F20.	[244]

The anti-amyloidogenic mechanism of DXC against two Aβ fibril polymorph forms, S-shaped and LS-shaped, was studied using accelerated MD (Table 3) [244]. It was found that the S-shaped pentameric fibril was partially destabilized, affecting the hydrophobic core consisting of residues N15–A30. Three main binding sites (BSs) were identified. The first was located near the side chain of M35, oriented toward the outer face of the fibril, where DXC interacted with the fibril backbone via hydrogen bonds and with M35 side chains through hydrophobic contacts. The second binding site was located between I32 and L34 on the solvent-exposed face of the larger hydrophobic core (N15–L34), where DXC formed hydrophobic contacts with the I32 and L34 side chains, as well as hydrogen bonds with the fibril backbone. The third binding site was identified between residues L17 and F19 at the solvent-accessible region of the hydrophobic core (N15–L34), where DXC was stabilized through hydrophobic interactions with L17 and F19. For the second fibril form, these sites are hidden; therefore, two different BSs were identified. The first was near the N-terminal of the LS-shaped fibril, where DXC engaged in polar interactions with E1 and hydrophobic contacts with V39 and I41. The second binding site consisted of residues K16, V18, and F20.

## 8. Mechanisms of Aβ Inhibition in Drug Discovery

Identifying Aβ inhibitors through structure-based drug design (SBDD) using molecular docking and molecular dynamics simulations is highly challenging. Typically, virtual screening through molecular docking, pharmacophore searches, or similarity searches can uncover potential novel Aβ inhibitors. Subsequent MD simulations can further strengthen these predictions, while in vitro and in vivo assays can validate them. Recently, a rapid in vivo pipeline for screening small-molecule inhibitors of Aβ aggregation was introduced [245]. Targeting fibrillogenesis is difficult due to its inherent complexity; the wide range of monomeric, dimeric, and oligomeric conformations with multiple binding sites; and the potential for false-positive predictions from computational approximations. However, a promising example was reviewed here [222]. Effective inhibitors must disrupt key fibril-stabilizing interactions or those essential for aggregation, as discussed in Section 3. Usually, the main affected secondary structure is the β-sheet content, an outstanding mark of Aβ oligomeric structures.

Moreover, both SBDD techniques and ligand-based drug design (LBDD) methods (a recent comprehensive review on the LBDD QSAR approach was published by us [246] and can be applied to known Aβ inhibitors) can be effectively employed in drug design for hit-to-lead optimization, based on a validated protocol, model, or pipeline of known Aβ inhibitors. These predictions can then be experimentally validated.

Mechanisms of Aβ inhibition can be categorized into the three main types: (1) Early-stage inhibition—This targets primary nucleation, i.e., the process of dimerization, where a small ligand hinders the interaction between two monomers. This interference can lead to an irregular or disordered nucleus or even prevent nucleation entirely (see Figure 1). (2) Middle-stage inhibition—This targets the formation of oligomers by interfering with the growth of pre-existing soluble low-molecular-weight oligomers (LMWO), such as dimers, trimers, and tetramers, which act as nucleation sites (see Figure 1). This mechanism can also affect secondary nucleation. (3) Disruption of existing oligomers—This strategy destabilizes already-formed LMWO or high-molecular-weight oligomers (HMWO), leading to the formation of irregular nuclei and non-fibrillar LMWO (see Figure 1). As a result, the aggregation process is diverted off-pathway, potentially yielding irregular β-aggregates or even complete disaggregation.

Unveiling the precise mechanisms of Aβ inhibition can be achieved through well-planned and properly conducted MD simulations, as outlined in the next section. Furthermore, these findings can be validated through in vitro and/or in vivo experiments and may also help guide future studies to confirm the proposed mechanisms.

## 9. Challenges and Perspectives in MD Simulations

The complexity of fibrillization leads to fibrils with diverse morphologies, further complicating the process of uncovering and gaining deep insights into the inhibition mechanisms of small molecules. Additionally, selecting force fields that best mimic the fibrillization process, along with an appropriate water model, is crucial. A third challenge lies in the availability of computational resources. To achieve a comprehensive and optimal understanding of the inhibition mechanisms of a given anti-amyloidogenic inhibitor using conventional MD simulations, the following key considerations should be taken into account: (1) The full length of the peptide should be considered, regardless of whether the simulation is performed on a monomeric peptide or a later-stage fibrillization unit such as a dimer, tetramer, or pentamer. (2) Simulations on protofibrils should be conducted for different morphological forms, such as U-, S-, and SL-shaped fibrils, to gain a thorough understanding of all currently known fibril structures. (3) Careful and appropriate selection of force fields for MD simulations is essential, and utilizing multiple force fields is preferable and recommended. (4) The water model should be carefully chosen to align with the selected force field for consistency and accuracy. (5) The molar ratio of the inhibitor to the peptide should be consistent with experimental data and, if possible, should mimic brain concentrations. Multiple molar ratios should also be considered. (6) In addition to studying the inhibitor of interest, a positive and negative control inhibitor, as well as a simulation without any inhibitor, should be included using the same MD simulation protocol. Additionally, the protonation state of the molecules should be considered at physiological pH. (7) The length of production dynamics should be sufficiently long to capture relevant structural changes, depending on computational resources and simulation time. (8) To gain a deeper understanding of the inhibition mechanism of a given anti-amyloidogenic compound, a comprehensive analysis should be performed, considering key stabilizing interactions of specific (proto-)fibril forms as well as the critical protein secondary structure element for fibril formation—the cross β-sheet content. (9) Finally, multiple simulation runs with different initial velocities should be performed to generate more reliable and statistically representative trajectories.

## Data Availability

Not applicable.

## Abbreviations and Acronyms

aa	amino acids
AD	Alzheimer’s disease
AICD	APP intracellular domain
aMD	accelerated molecular dynamics
APP	amyloid-β (A4) precursor protein
ATP	adenosine triphosphate
BACE1	β—secretase 1, β- site amyloid precursor protein (APP) cleavaging enzyme 1
BBB	blood–brain barrier
BS	binding site
CBX	Carbenoxolone
CG	coarse-grained
CGM	coarse-grained model
cMD	classical/conventional molecular dynamics
CTF	C-terminal fragment of APP
DA	dopamine
FAD	familial Alzheimer’s disease
FF	force field
IDP	intrinsically disordered proteins
LMW	low molecular weight
LMWO	low molecular weight oligomers
MD	molecular dynamics
MEL	melatonin
NE	norepinephrine
NMR	nuclear magnetic resonance
PPF	Propafenone
RE	replica exchange
REM	replica exchange method
REMD	replica exchange molecular dynamics
REXAMD	replica exchange accelerated molecular dynamics
ROS	reactive oxygen species
SAD	sporadic Alzheimer’s disease
SER	serotonin
SPC	simple point charge water model
STDR	simulated tempering distributed replica
TIP3P	transferable intermolecular potential 3-point water model
TMD	transmembrane domain
